# Home Health Agencies With More Socially Vulnerable Patients Have Lower Experience-of-Care Ratings

**DOI:** 10.1093/geroni/igab046.2053

**Published:** 2021-12-17

**Authors:** Jinjiao Wang, Meiling Ying, Yue Li

**Affiliations:** University of Rochester, Rochester, New York, United States

## Abstract

Little is known about the disparities in patient experience of home health (HH) care related to social vulnerability. This study examined the relationships of patient Medicare-Medicaid dual eligible status and race and ethnicity with patient experience of HH care. We analyzed national data from the Home Health Care Consumer Assessment of Healthcare Providers and Systems (HHCAHPS), Outcome and Assessment Information Set, Medicare claims and Area Health Resources File for 11,137 Medicare-certified HH agencies (HHA) that provided care for Medicare beneficiaries in 2017. Patient-reported experience of care star ratings (1-5) in HHCAHPS included 3 domains (professional care delivery, effective communication, and specific issues in direct patient care) with each dichotomized into high (4-5) and low (1-3) experience of care. The proportion of patients with dual eligibility and the proportion of racial/ethnic minorities were summarized at the HHA level. HHA with higher proportion of dual eligible patients were less likely to have high experience of care rating in professional care delivery (smallest Odds Ratio [OR]=0.514; 95% CI: 0.397, 0.665; p<0.001), effective communication (smallest OR=0.442, 95% CI: 0.336, 0.583; p<0.001), and specific direct care issues (smallest OR=0.697, 95% CI: 0.540, 0.899; p=0.006). HHA with higher proportion of racial/ethnic minorities were also less likely to have high patient experience of care rating across all three domains (smallest OR=0.265, 95% CI: 0.189, 0.370; p<0.001). Disparities in patient experience of HH care exist and they are associated with low income and racial/ethnic minority status, indicating substantial unmet needs among these socially vulnerable patients.

